# The RaDiCEA Project: cost of illness (COI) analysis applied to Cryopyrin Associated Periodic Syndromes (CAPS)

**DOI:** 10.1186/1546-0096-13-S1-P186

**Published:** 2015-09-28

**Authors:** O Della Casa Alberighi, L Trieste, L Accame, V Lorenzoni, F Pierotti, S Federici, M Gattorno, P Quartier, P Duong Ngoc, N Cabrera Rojas, A Martini, G Turchetti

**Affiliations:** 1 Istituto di Ricovero e Cura a Carattere Scientifico (IRCCS) Giannina Gaslini, Unità di Farmacologia Clinica e Sperimentazioni Cliniche, Direzione Scientifica, Genova, Italy; 2 Scuola Superiore Sant'Anna, Institute of Management, Pisa, Italy; 3 Istituto di Ricovero e Cura a Carattere Scientifico (IRCCS) Giannina Gaslini, Pediatria II, Reumatologia, Genova, Italy; 4 Assistance Publique-Hopitaux de Paris Hopital Necker,, Paediatric Immunology, Haematology and Rheumatology Unit, Paris, France

## Introduction

When a disease affects only a few individuals in each country (ultra-orphan disease), it can be very hard to establish the costs of illness (COI) and the cost-effectiveness (CE) of treatments (ultra-orphan drugs). Conventional methods for COI and CE of drugs for common conditions do not apply, and additional factors need to be considered. As expensive medications (biologicals) show promising results, it becomes crucial to have detailed information on as many patients as possible.

## Objectives

This international, multicenter, longitudinal observational COI and CE study will evaluate the burden of CAPS in terms of direct and indirect costs and quality of life (QoL). The COI analysis implies the identification, quantification and evaluation of resources from a societal perspective.

## Materials and methods

In the frame of the EuroFever registry (http://www.printo.it/eurofever/), 8 Centers of reference for autoinflammatory diseases in Italy and France (sentinel countries) are currently enrolling all consenting/assenting CAPS adult patients and children with the indication for either the use of an anti IL-1 agent or other than anti IL-1 therapies (NSAIDs, systemic corticosteroids, immunosuppressive drugs), and compliant to the study. To perform the COI analysis, clinical and economic data are collected at country-specific- and general levels, both retrospectively (since disease onset) and prospectively (from study enrollment to the last visit) by mean of general questionnaires administered to patients or their proxy on direct medical and non-medical costs, and indirect costs of productivity loss. QoL is prospectively measured using EQ5D to estimate utility score and calculate QALY.

## Results

COI analysis results based on the Italian and French Healthcare Systems will test the robustness of the proposed methodology, assumptions and source of data before a generalization to other countries. To properly value the used resources, country-specific questionnaire administered to the budget and management control officer at each center will collect information about the healthcare system type, hospitalization reimbursement system, out of hospitalization drug payment, payment of special schools and devices, application of exemption, disability pension and/or allowance for caregivers. All costs will be referred to a common base year and adjusted to eliminate the effect of inflation. The differences in price levels between countries will be eliminated applying the Purchase Power Parities using Euros as the reference base.

## Conclusion

The ERANET-PRIOMEDCHILD RaDiCEA Project (No. 40-41800-98-007) will develop a model to evaluate costs and long-term benefits in an ultra-orphan group of diseases such as CAPS. The same model may be used in other very rare disorders.

